# Functional Connectivity Density for Radiation Encephalopathy Prediction in Nasopharyngeal Carcinoma

**DOI:** 10.3389/fonc.2021.687127

**Published:** 2021-07-12

**Authors:** Lin-Mei Zhao, Ya-Fei Kang, Jian-Ming Gao, Li Li, Rui-Ting Chen, Jun-Jie Zeng, You-Ming Zhang, Weihua Liao

**Affiliations:** ^1^ Department of Radiology, Xiangya Hospital, Central South University, Changsha, China; ^2^ School of Psychology, Shaanxi Normal University, Shanxi Provincial Key Research Center of Child Mental and Behavioral Health, Xi’an, China; ^3^ Department of Radiation Oncology, State Key Laboratory of Oncology in South China, Collaborative Innovation Center for Cancer Medicine, Sun Yat-sen University Cancer Center, Guangzhou, China; ^4^ State Key Laboratory of Oncology in South China, Collaborative Innovation Center for Cancer Medicine, Sun Yat-sen University Cancer Center, Guangzhou, China; ^5^ Department of Radiology, Hunan Children’s Hospital, Changsha, China

**Keywords:** radiotherapy, nasopharyngeal carcinoma, magnetic resonance imaging, machine learning, follow-up

## Abstract

The diagnostic efficiency of radiation encephalopathy (RE) remains heterogeneous, and prediction of RE is difficult at the pre-symptomatic stage. We aimed to analyze the whole-brain resting-state functional connectivity density (FCD) of individuals with pre-symptomatic RE using multivariate pattern analysis (MVPA) and explore its prediction efficiency. Resting data from NPC patients with nasopharyngeal carcinoma (NPC; consisting of 20 pre-symptomatic RE subjects and 26 non-RE controls) were collected in this study. We used MVPA to classify pre-symptomatic RE subjects from non-RE controls based on FCD maps. Classifier performances were evaluated by accuracy, sensitivity, specificity, and area under the characteristic operator curve. Permutation tests and leave-one-out cross-validation were applied for assessing classifier performance. MVPA was able to differentiate pre-symptomatic RE subjects from non-RE controls using global FCD as a feature, with a total accuracy of 89.13%. The temporal lobe as well as regions involved in the visual processing system, the somatosensory system, and the default mode network (DMN) revealed robust discrimination during classification. Our findings suggest a good classification efficiency of global FCD for the individual prediction of RE at a pre-symptomatic stage. Moreover, the discriminating regions may contribute to the underlying mechanisms of sensory and cognitive disturbances in RE.

## Introduction

Nasopharyngeal carcinoma (NPC) is a malignancy stemming from the nasopharyngeal epithelium, and more than 70% of all new cases are confirmed in the east and southeast Asia (1). Recently, the optimization of radiotherapy and chemotherapy strategies has considerably improved disease control and survival (2). Nevertheless, some long-term treatment-related complications still seriously affected the patients’ quality of life. This is especially true of radiation encephalopathy (RE), which has captured the attentions of clinicians and researchers alike for its deteriorating neuropsychiatric symptoms, sometimes even causing death (3). Early intervention has been reported to improve patient prognosis; however, existing conventional magnetic resonance imaging (MRI) techniques can only discern RE at the irreversible stage (4). The early identification or individualized prediction of RE is therefore crucial for improving quality of life and prognosis in patients with RE.

The advent of other neuroimaging techniques has enabled the earlier detection of radiation-induced alterations in patients with NPC (5–7). The neuroimaging index reflects a disease-specific pathological or neurophysiological property and may even be an early biomarker of such alterations. For morphology, one gray matter morphology-based study has suggested that cortical surface area might be a morphological marker of patients with early-stage RE (8). In addition, a white matter connectivity-based structural network study revealed a network-level reorganization in the late-delayed stages of RE (9). However, most studies have mainly focused on the differences at group levels; far less attention has been paid to the potential value of individual levels.

With the emergence of multivariate pattern analysis (MPVA), the individual recognition of neurological diseases is possible. Several recent reports about the individualized prediction of RE have been promising. For example, a machine-learning study used texture features to develop radiomics models for the dynamic prediction of RE (10). However, these texture features were from the medial temporal lobe, and information from outside the medial temporal lobe was insufficiently investigated. Another recent support vector machine (SVM) study based on white matter integrity reported good abilities for diagnoses in different periods of RE (11). Unfortunately, the above discriminative power of gray matter-derived features has been largely overlooked.

A recent study has demonstrated that functional parameters are altered earlier and are more vulnerable than those that reflect structural integrity (5), suggesting that aberrance in functional domains may play a critical role in the pathogenesis of RE. Furthermore, using resting-state functional MRI (rs-fMRI), the fact that neurophysiological characteristics of neuroimaging function alterations in RE involved the whole brain (12) makes large-scale functional evaluation notable. Functional connectivity density (FCD) allows researchers to evaluate the whole-brain functional brain connectivity patterns at the voxel level (13). It can reflect the early patterns of disease-specific neuronal activity changes (14–16). To the best of our knowledge, FCD has not yet been used to predict RE at the pre-symptomatic stage. Therefore, the combination of FCD and machine learning strategies in the present study may contribute to a better understanding of the pathological mechanisms of RE and aid in its early prediction.

## Materials and Methods

### Study Design and Subjects Enrollment

**Figure 1:**
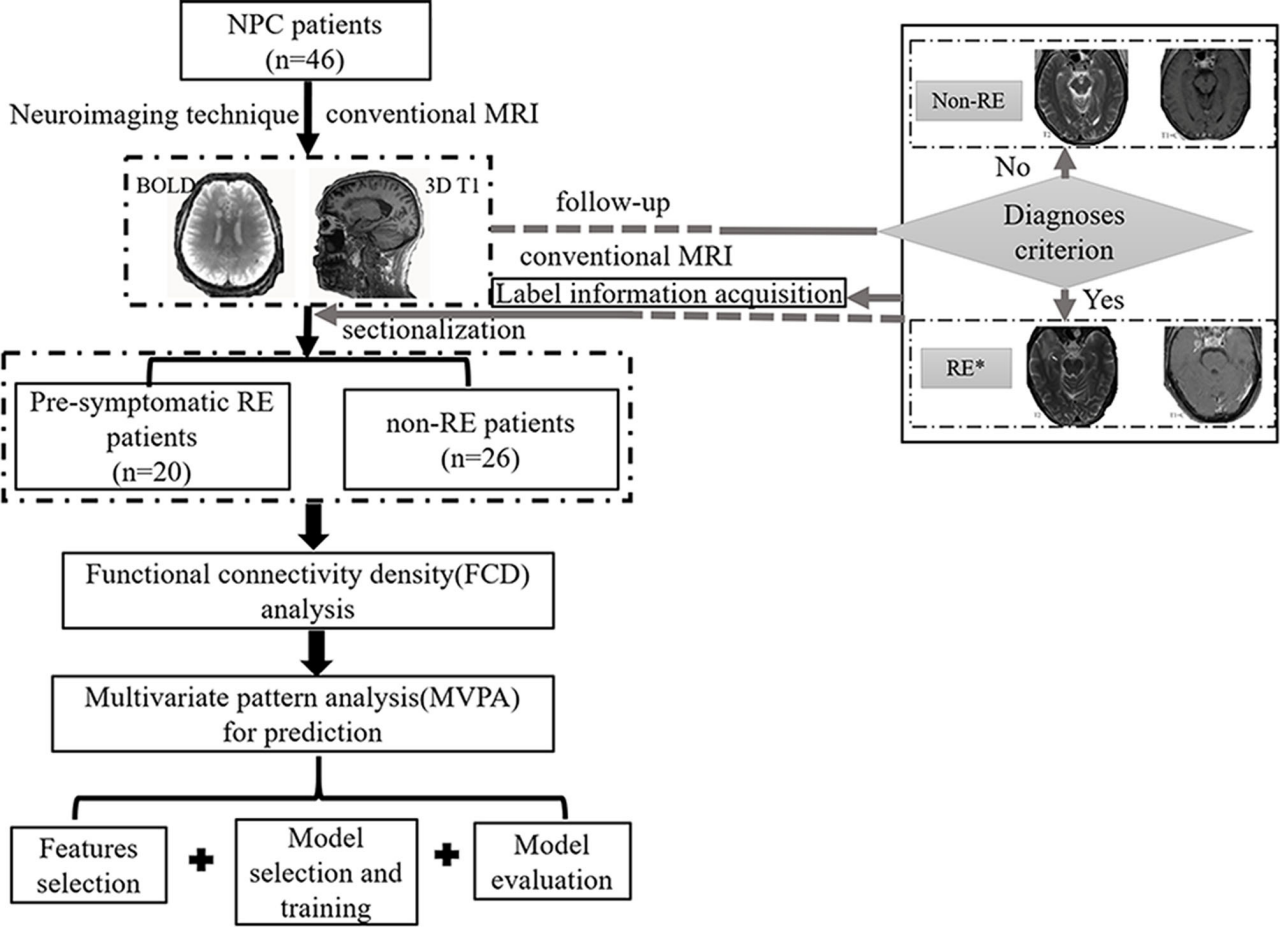
The flowchart of this study. *, patients with radiation encephalopathy were confirmed by Merritt’s Neurology; NPC, nasopharyngeal carcinoma; RE, radiation encephalopathy; BOLD, blood oxygen level-dependent; T2, T2 weighted image; T1+C, T1 weighted image + contrast.

We developed the MVPA from a cohort of 46 NPC patients. All participants were right-handed and had pathologically confirmed NPC. Other specific inclusion criteria were as follows: 1) aged between 20 and 60 years with over 6 years of education; 2) NPC patients who underwent radiotherapy within the previous 6 months; 3) no abnormalities of RE; and 4) no presentation of any other intracranial or central nervous system diseases. Patients were excluded if they had a consciousness disorder, central nervous system disease, or any other disease. All patients were treated with radiotherapy before the study using either two-dimensional radiation therapy (2DCRT) or intensity-modulated radiation therapy (IMRT). To control the confounding effect of chemotherapy on the FCD changes, all the enrolled patients treated with chemotherapy had balanced between group clinical stages, chemotherapy mode, regimens and chemotherapy type by reading their MR images and medical records (**Table 1**) (8). The detailed information of chemotherapy (such as chemotherapy agents, dose for each agent, time for medication administration, number of courses, and duration) for patients with NPC in this study could be obtained in **Supplementary Materials** **(** **Table S1**). The NPC patients were then divided into subgroups based on whether or not their conventional images meet the RE diagnoses criterion (3) during the follow-up (72 ± 8 months). Specifically, the subsequent neuroimaging analysis was based on original data rather than followed-up data. The exact procedures are shown in the overall flowchart in **Figure 1**. Informed consent was obtained from all subjects, and the ethics committee approved the study before its execution.

**Table 1:** Demographic and clinical characteristics.

Characteristics	NPC patients followed-up with RE (n = 20)	NPC patients followed-up without RE (n = 26)	*P*-value
**Age (year)**	45.10 ± 9.63	44.54 ± 11.24	0.86
**Gender (male/female)**	15/5	17/9	0.53
**Clincial stage**			0.883
I/II, n	5 (10.87%)	7 (15.22%)	
III/IV, n	15 (32.61%)	19 (41.30%)	
**Teatment option**			0.289
Radiotherapy only, n	2 (4.35%)	7 (15.22%)	
Radio-chemotherapy, n	18 (39.13%)	19 (41.30%)	
**Radiotherapy time (month)**	22.70 ± 28.43	32.54 ± 27.43	0.89
**Chemotherapy mode for patients treated with radio-chemotherapy**			0.604
Neoadjuvant and concomitant chemotherapy, n	16 (43.24%)	18 (48.65%)	
Others, n	2 (5.41%)	1 (2.70%)	
**Chemotherapy regimens for patients treated with radio-chemotherapy**			0.447
TPF/TP/PF, n	13 (35.14%)	16 (43.24%)	
GP, n	5 (13.51%)	3 (8.11%)	
**Chemotherapy type**			NA
Target-directed chemotherapy, n	0 (0)	0 (0)	
Conventional chemotherapy, n	18 (48.65%)	19 (51.35%)	

Note: NPC, nasopharyngeal carcinoma; RE, radiation encephalopathy; TPF, docetaxel, cisplatin and fluorouracil; TP, docetaxel and cisplatin; PF, cisplatin and fluorouracil; GP, gemcitabine and cisplatin; NA, not available. Clinical stage were obtained according to the 7th edition of the UICC/AJCC (2009) TNM. Stage I: T1N0M0; Stage II: T0-1N1M0 and T2N0-1M0; Stage III: T0-2N2M0 and T3N0-2M0; Stage IV: T4N0-2M0,or N3 or M1.

### MRI Acquisition

MRI images were collected using a 3.0-T MRI scanner (MAGNETOM Tim Trio, Siemens, Germany). Functional imaging data were generated from echo-planar imaging sequences, and the main parameters were as follows: repetition time = 2,400 ms, echo time = 30 ms, matrix size = 64 × 64, flip angle = 90°, number of timepoints = 240, field of view = 230 mm × 230 mm, and 40 axial slices. During the rs-fMRI sessions, participants were asked to keep their eyes closed, without falling asleep or thinking of anything. Three-dimensional T1-weighted magnetization-prepared rapid acquisition with gradient echo sequences were taken as follows: 176 sagittal slices overall, voxel size = 1.0 mm× 1.0 mm× 1.0 mm, slice thickness/gap = 1.0/0 mm, matrix size = 256 × 256, field of view = 256 mm × 256 mm, repetition time = 2,300 ms, echo time = 2.98 ms, flip angle = 9°. Routine sequences were scanned to ensure a diagnosis of RE and exclude other diseases.

### FCD Analysis

The rs-fMRI data were first preprocessed using the Data Processing & Analysis for Brain Imaging (DPABI) toolbox (https://rfmri.org/dpabi) (17), and the initial 10 volumes were removed. Next, slice-timing, realignment, spatial normalization, regression of nuisance covariance, and temporal filtering steps were performed. The preprocessed data were then used for FCD mapping calculation with an in-house script in MATLAB according to the methods described by Tomasi and Volkow (13). FCD mapping was used to compute the global FCD (gFCD) as well as the local FCD (lFCD) in identified distributions of hubs in the brain (18). Further details are documented below.

#### Data Preprocessing

A toolbox for Data Processing & Analysis for Brain Imaging (19) (DPABI; https://rfmri.org/dpabi) pipeline was used to preprocess the rs-fMRI data, as follows: (1) The first 10 volumes were removed to adapt subjects to the scanning environment and lower the magnetization equilibrium; (2) Slice-timing correction: the proper slice order and reference order were selected; (3) Realignment: the time series of each subject were realigned using a linear transformation with six-parameter (rigid-body) and head motion correction [translational displacement [x, y, or z directions] <2.0 mm, or maximum rotation <2.0° (20)] were carried out; individual three-dimensional T1 images were subsequently co-registered to mean resting images using linear transformations (6° of freedom) without re-sampling and were later segmented into the different components of gray matter, white matter and cerebrospinal fluid; (4) Spatial normalization was performed using the DARTEL tool (21) for transformations from the individual native space to the MNI space (3 mm × 3 mm × 3 mm voxel size); (5) Linear regression was performed for nuisance covariates, including head motor parameters from the realignment step (the Friston 24-parameter model), global mean signals, white matter, and cerebrospinal fluid signals; and (6) All available images were temporally filtered with a 0.01–0.08 Hz bandpass to minimize the effects of high-frequency physiological noises and low-frequency drift.

#### FCD Calculation

The FCD calculation was restricted to voxels within the gray matter mask, which was predefined through tissue with probabilities of more than 20% in the gray matter probability template (22). Pearson correlation coefficient at the threshold of R >0.6 determined the functional connectivity between voxels. We selected this threshold of 0.6 because of its relatively high specificity and sensitivity (14). The related scripts were showed in **Supplementary Material**.

#### lFCD

The lFCD of a given voxel (x0) was computed using a “growing” algorithm. Specifically, the number of functional connections for any given voxel (xn) and its adjacent voxels (xni) was calculated. First, the time series of a given voxel (x0) and its adjacent voxels (xi) were calculated using Pearson correlation analysis. Each xi was added to a cluster only when the Pearson correlation coefficient was larger than the threshold (Ri0 >0.6). Next, the Pearson correlation for a time-varying series between x0 and a voxel (xj) adjacent to xi was also evaluated; similarly, each xj was added to the aforementioned cluster when Rj0 >0.6. This process was repeated in an iterative way for all other voxels (N − 1) that were adjacent to voxels in the aforementioned cluster and functionally connected to x0, until no fresh voxels were able to be added to the cluster. The lFCD at x0 was defined as the number of units in the local functional connectivity cluster, k(x0). After finishing this process for a given voxel (x0), the calculation was initiated for a different given voxel. This calculation was performed for all N voxels.

#### gFCD

The gFCD for a given voxel x0 was defined as the global number of functional connections, k(x0), between x0 and all other global voxels. This calculation was also iterated for all given voxels (N) in the global brain and underwent the operation of N × (N − 1)/2 correlations.

All FCD maps were normalized to the average FCD of individual whole brains (FCD normalized [x, y, z] = FCD [x, y, z]/mean FCD [k0]). Finally, all FCD maps were spatially smoothed using an 8 mm full-width at half-maximum (FWHM) Gaussian kernel before the subsequent analysis.

### Statistical Analysis

Demographic information and FCD maps were compared between pre-symptomatic RE and non-RE groups. Unpaired t-tests and χ2 tests were used to analyze demographic information. Unpaired t-tests were conducted to compare FCD maps with age, gender, and years of education as covariates. P <0.05 was considered to indicate statistical significance.

### SVM Analysis Using FCD

A linear kernel SVM algorithm was applied based on Pattern Recognition for Neuroimaging Toolbox (PRoNTo version 2.0, http://www.mlnl.cs.ucl.ac.uk/pronto) to estimate the underlying brain regions that most contributed to classifying pre-symptomatic RE *versus* non-RE subjects (23). The central bodies of the SVM method were briefly concluded as follows: 1) features extraction and selection, 2) discriminative regions selection, 3) the SVM classifier model training using the training data, and 4) evaluation of the performances of the SVM model using the evaluation data.

In this present study, feature selection consisted of the FCD values that were expected to show statistical significance between the two groups. The procedures aforementioned above were automatically processed using Prepare feature set pipeline of PRoNTo.

The leave-one-out cross-validation method was applied to validate the SVM classifier’s validation. Each time, feature selection was conducted using the training data to avoid circularity effects. The training data in this step involved (n − 1) subjects, and the excluded single subject was used to test the generalization ability (i.e., the ability to reliably classify new samples). These above steps were repeated n times (n = the number of subjects) until the classifier generalizability was unbiased. The process was automatically computed using the ‘specify model’ pipeline of PRoNTo.

Classifier performance was evaluated by its accuracy, sensitivity, specificity, and area under the receiver operating characteristic operator curve (AUC), with the procedure repeated for each pair of the subject. Furthermore, a 5,000 times non-parametric permutation test performed the evaluation, with corrected P <0.05 denoting significance in this evaluation. The aforementioned procedures were selected and automatically computed using the ‘run model’ and ‘display results’ pipelines of PRoNTo.

The ‘compute weights’ and ‘display weights’ pipelines were also run using PRoNTo. These pipelines produced the voxel weight vectors and a list of regions in descending order according to their contributions to the classification model. The voxel weight vectors were subsequently converted to a map, which was visualized using the BrainNet Viewer (24).

## Results

### Demographic and Clinical Characteristics

The demographic and related clinical results are displayed in **Table 1**. The two groups were matched for age, gender, clinical stage, treatment options, and therapy time. Chemotherapy parameters such as chemotherapy mode, regimens, and types were not significantly different between the two groups (*P >*0.05).

### Classification Results

**Figure 2:**
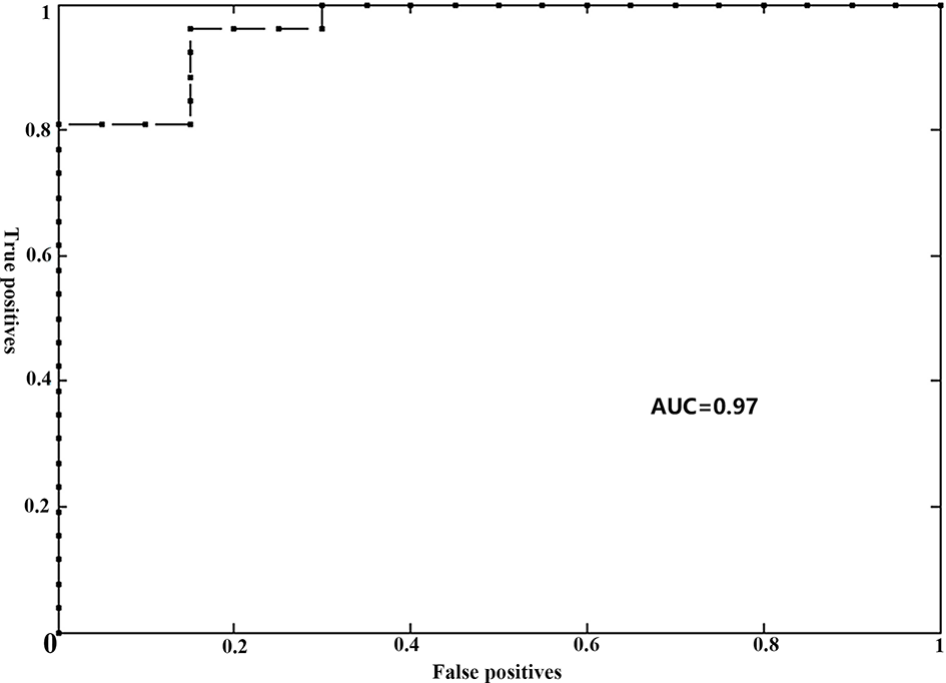
Receiver operator curve (ROC) for individual prediction of RE at the pre-symptomatic stage in patients with NPC. RE, radiation encephalopathy; NPC, nasopharyngeal carcinoma; AUC, the area under the curve.

The gFCD was significantly different between the two groups (*P* <0.05), while the lFCD was not. We therefore selected the gFCD as the feature for classification. The linear SVM analysis predicted a diagnosis of RE using gFCD with a total accuracy of 89.13% and a balanced accuracy of 88.08% (sensitivity of 80.00%, and a specificity of 96.15%). The receiver operating characteristic (ROC) curve and AUC were also plotted (**Figure 2**). The AUC was 0.97, and permutation tests for the AUC revealed statistical significance.

### Brain Weighted Location Model

**Figure 3:**
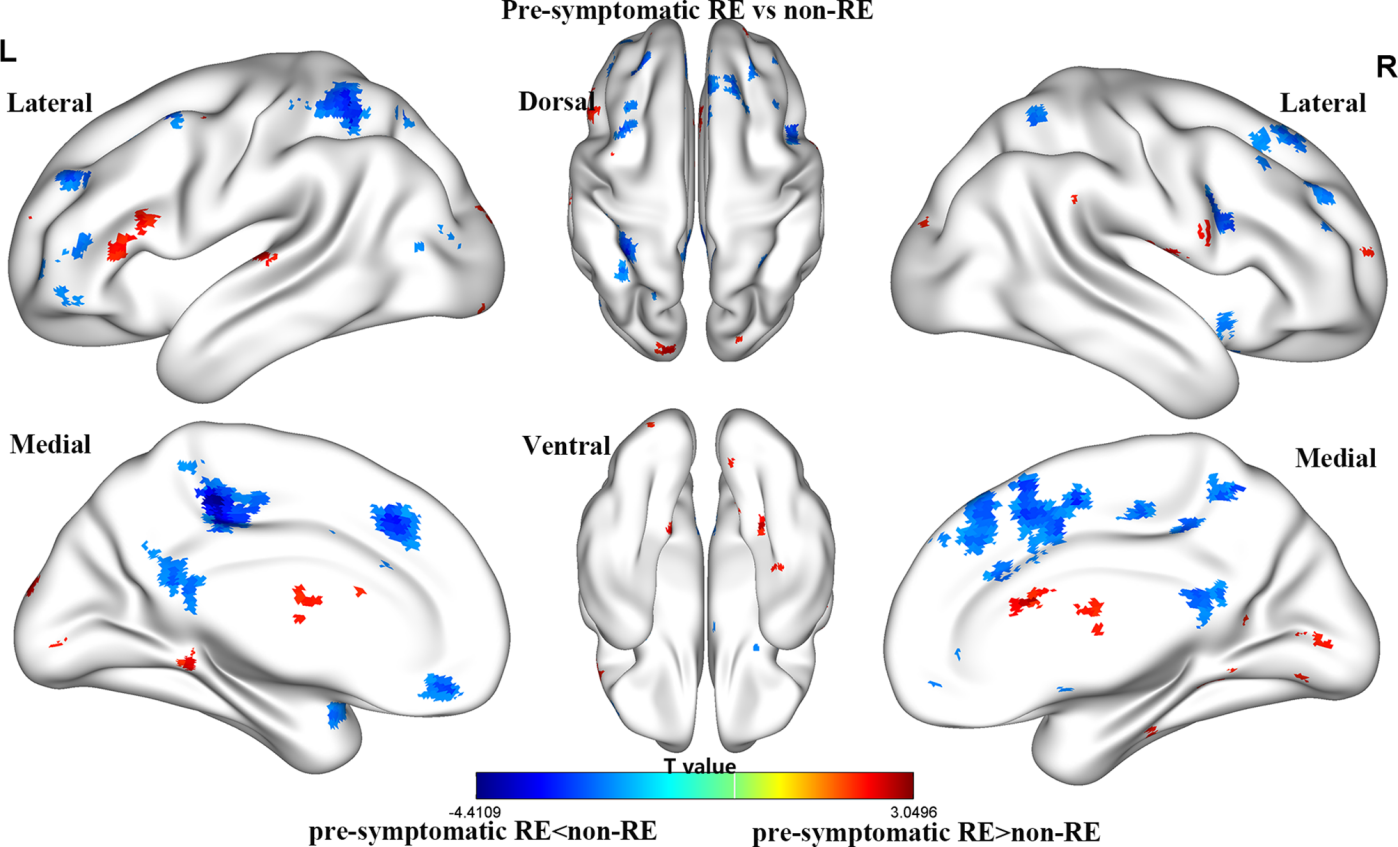
The brain maps of pre-symptomatic RE and non-RE based on gFCD at the voxel level. RE, radiation encephalopathy; NPC, nasopharyngeal carcinoma; FCD, functional connectivity density.

**Figure 4:**
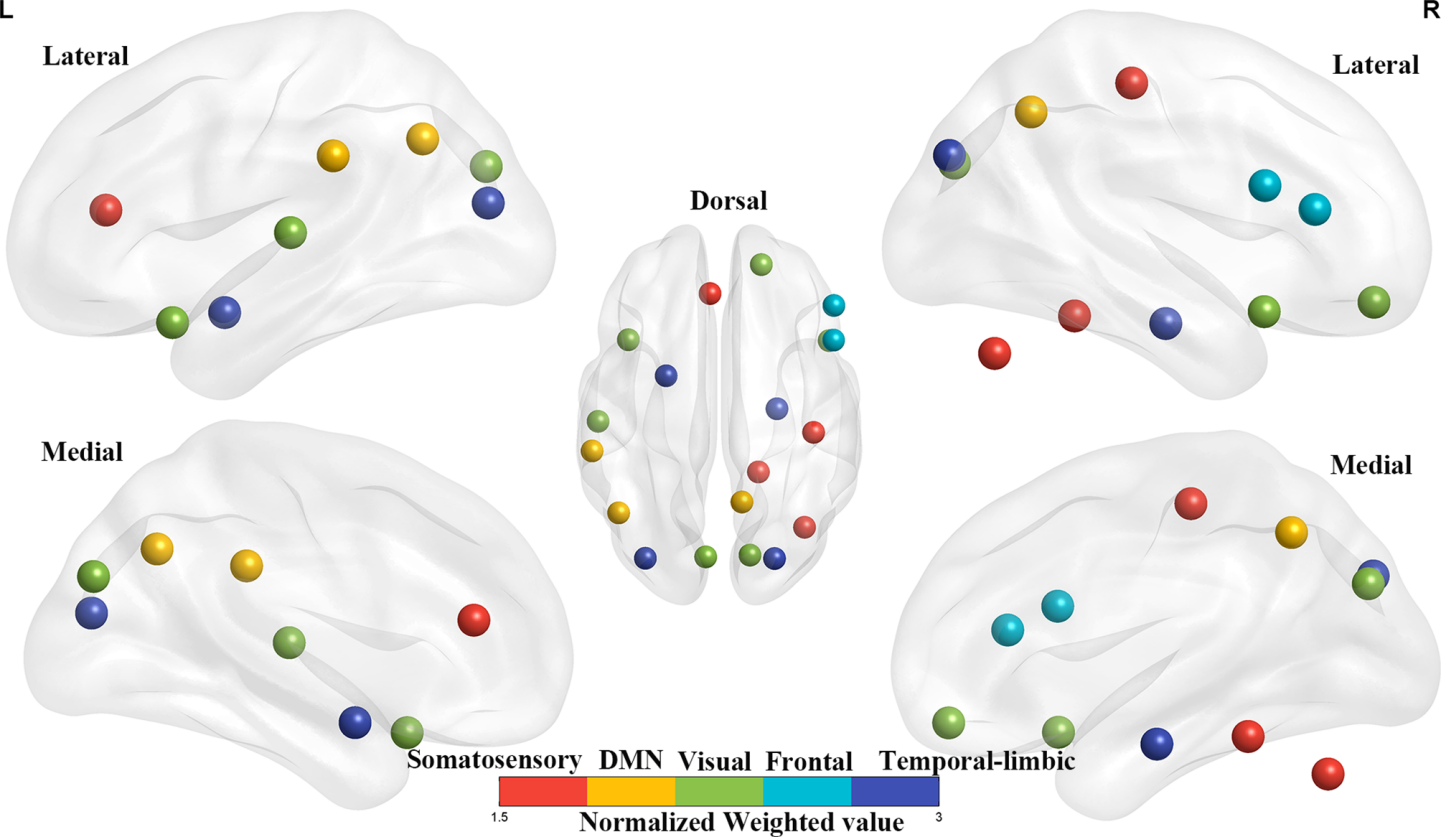
The top 20 weighted brain regions’ distribution spatially. The color bar denotes the percentage of total normalized weights that each brain region explains. DMN, default mode network.

For the gFCD, we presented the weighted voxel distribution to classify between RE patients and non-RE patients (**Figure 3**). The top 20 spatial distribution in terms of normalized weights per region was revealed to 43.29% of the predictive weights (**Table S2**). These regions identified (**Figure 4**) through weighted landmarks mainly included the bilateral temporal pole and cuneus. Unilateral regions of the left hemisphere covered the superior temporal cortex, middle occipital cortex, amygdala, angular and supramarginal cortex, and anterior cingulum cortex (ACC). In contrast, regions of the right hemisphere consisted of the opercular and triangular parts of the inferior frontal cortex, the parahippocampus, and the postcentral and precuneus gyri, together with part of the right regions of the cerebellum and its crus.

## Discussion

This was the first study to examine FCD alterations between pre-symptomatic RE and non-RE NPC patients, which were then used to predict the occurrence of RE using a machine learning approach. The FCD analysis revealed that gFCD was altered in patients with pre-symptomatic RE. Upon closer inspection of these results, we revealed that brain regions with gFCD abnormalities were mainly found in the bilateral temporal lobe, as well as in regions involved in the visual pathway, the somatosensory system, and the default mode network (DMN). Moreover, gFCD alterations in these brain regions were able to predict RE in high performance with an accuracy of 89.13%. This finding suggests that gFCD may be a novel imaging biomarker for the early detection of RE, which may contribute to a better understanding of its pathogenesis.

### Classification Interpretation

In this current study, the predictive performance of the model was generally favorable, as evidenced by its accuracy of 89.13% and an AUC of 0.97. The prediction efficiency observed in our study was also higher than those of previous MVPA studies (10, 11, 25). For example, using functional connectivity as a feature, Ma et al. reported an accuracy of 81.36% for differentiating patients with and without RE (25). Another MVPA study used fractional anisotropy or white matter connections to identify the individuals at a high risk of RE, with a maximum accuracy of 84.5% (11). Furthermore, based on conventional MRI, a recent radiomics model study reported a maximum AUC of 0.83 for predicting RE (10). We speculated that different feature selection and/or modeling strategies might be responsible for the inconsistencies in the prediction accuracies for RE among these studies. Given that distinct features may reflect a specific physiological process, our findings of a better performance in the prediction of RE using FCD suggested that FCD may be a sensitive neuroimaging biomarker for reflecting the radiation-induced functional impairments.

### Brain Weight Location Model

Notably, we found that gFCD, rather than lFCD, made a substantial contribution to the predictive model for the early diagnosis of RE. Our results are partially supported by those of previous studies, which demonstrated that gFCD is more sensitive to individual differences than lFCD in terms of functional connectivity (18). It has been well documented that altered gFCD is linked to functional deficits in multiple domains [such as attention (15), cognition (26), memory, and visual perceptual (16)], which are all common clinical symptoms in patients with RE (3, 27). Although the potential factors secondary to pathological alterations of radiation-induced vascular endothelial cell injury and vascular stenosis may lead to FCD abnormalities (28), the exact neural mechanisms underlying the observed patterns of FCD changes remain unclear.

The current study revealed that gFCD in the temporal regions and cuneus had good identification efficiency in patients with RE. These results were not surprising; the temporal areas [including the medial and inferior aspects (29)] are located in the radiation field and are thus vulnerable to injury (30). Recently, several neuroimaging studies (7, 8) have reported structural alterations in the temporal lobe following radiation therapy (31). For example, using voxel-based morphology (VBM) (31), decreased cortical volumes of the temporal regions were reported after radiation therapy in patients with NPC. One surface-based morphometry study reported increased cortical thickness of the left superior temporal gyrus (STG) in patients with NPC following radiation therapy (7). Another SBM study (32) revealed an increased cortical surface area in the temporal lobe and decreased cortical thickness in the bilateral temporal pole and STG. Aside from the structural evidence, our findings of altered gFCD in the temporal lobe are further supported by previously documented radiation-induced functional impairments (such as abnormal regional homogeneity (ReHo) and functional connectivity) in the temporal pole and STG (5, 12). Of note, the temporal gyrus and cuneus, where gFCD was altered in our study, can integrate visual information from the anterior visual pathway (33), whose lower stream (eyes lens, optic nerve, and optic chiasm) undergoes severe radiation-induced damage (34). We therefore speculated that the altered gFCD in the cuneus and temporal regions might reflect functional impairments in the anterior visual processing pathway (cuneus–temporal lobe loop). Furthermore, a previously reported increase in visual evoked potential latency and a decrease in the amplitude (35) of the anterior visual pathway of patients with RE may further support our hypothesis.

In our study, gFCD in the postcentral gyrus and ACC also contributed substantially to the early diagnosis of RE. Our findings are partially supported by several previous functional studies (6, 12), which reported increased ReHo in the postcentral gyrus and decreased FC in the postcentral gyrus and ACC. Furthermore, one SBM study provided structural evidence with cortical thickness abnormalities in the postcentral gyrus and ACC in patients with NPC after radiation therapy (7). Physiologically, the ACC receives inputs from the spinothalamic tract (36), which then projects to the postcentral gyrus, thus constituting the somatosensory pathway. One case report has also demonstrated that the injury of the spinothalamic tract can occur as a result of the primary brainstem injury (37), as the brainstem is located in the radiation field and receives a high radiation dose in patients with NPC (34). Taken together, the abnormal brain activity of the postcentral gyrus and ACC thus be a secondary response to the damaged sensory neural circuit in the brainstem. Moreover, sensory deficits, such as facial (38) and limb numbness or pain perception (39), that are observed in NPC patients after radiotherapy suggest that functional impairments occur in the sensory in the neural circuit.

We observed that the gFCD within the precuneus, supramarginal gyrus, and angular gyrus was crucial for predicting RE. The precuneus (32), and the inferior parietal cortex (supramarginal gyrus and angular gyri), are functionally connected and formed a resting-state brain network, known as the DMN. As has been reported, the DMN has self-referential, introspective-state functions, and processes an individual’s thoughts and feelings (40, 41). To date, many previous studies have identified structural and functional abnormalities in DMN-associated brain regions, such as decreased cortical thickness (7), the abnormal fractional amplitude of low-frequency fluctuations (fALFF) (5), ReHo, and FC (6, 12). Thus, together with the previous observations, our results indicated that the activity of DMN activity might be a potential neurological biomarker for radiation-induced cognitive impairments; however, this needs further investigation.

### Limitations

Some limitations were presented in this study. First, the study contained a relatively small series of patients because of the relatively low morbidity of RE as well as low patient compliance during follow-up. Although the current SVM algorithm was appropriate for a small sample size, future studies would benefit from a larger sample and would have a more stable predictive performance. We have thus started to create a larger RE database for further investigations. Secondly, the lack of any comprehensive assessments of cognitive function weakens the interpretability of our results. Future studies that use detailed cognitive scales will be indispensable for the validation of such an investigation. Thirdly, chemotherapy has been reported to exert side effects on the cerebral functional domain in patients with NPC following radiotherapy. We tried to control for the effects of confounding factors by keeping TNM stages and chemotherapy regimens consistent. However, further research is warranted to exclude the chemotherapy-related confounding effects on the radiation-induced functional impairments.

## Conclusions

In the current study, we analyzed FCD maps using a machine learning SVM algorithm to predict RE in NPC patients for the first time. The gFCD was revealed to have a good prediction efficiency. This finding provides insights into voxel-level cerebral information and suggests that gFCD might be a valid biomarker of RE. Furthermore, brain regions within the temporal pole and those involved in visual processing, the somatosensory system, and the DMN showed high discrimination, which may help to explain the sensory and cognitive disturbances that occur in RE.

## Data Availability Statement

The original contributions presented in the study are included in the article/**Supplementary Material**. Further inquiries can be directed to the corresponding authors.

## Ethics Statement

The studies involving human participants were reviewed and approved by the Ethics Committee of Xiangya Hospital, Central South University. The patients/participants provided their written informed consent to participate in this study.

## Author Contributions

L-MZ, Y-MZ, and W-HL designed and supervised this study. J-MG, LL, R-TC, J-JZ contributed to data acquisition, and Y-FK analyzed the data. L-MZ, Y-MZ, and W-HL wrote and revised the manuscript. All authors contributed to the article and approved the submitted version.

## Funding

This study was funded by the National Natural Science Foundation of China, Grant/Award Numbers: 82001784, 91959117, 82071894; The Youth Science Foundation of Xiangya Hospital, Grant/Award Number: 2019Q16; The Natural Science Foundation (General Project) of Hunan Province, Grant/Award Number: 2018JJ2271.

## Conflict of Interest

The authors declare that the research was conducted in the absence of any commercial or financial relationships that could be construed as a potential conflict of interest.

## Abbreviations

2DCRT, two-dimensional radiation therapy; ACC, anterior cingulum cortex; DPABI, Data Processing & Analysis for Brain Imaging; DMN, default mode network; fALFF, fractional amplitude of low-frequency fluctuations; g/lFCD, global/local functional connectivity density; IMRT, intensity-modulated radiation therapy; MPVA, multivariate pattern analysis; NPC, nasopharyngeal carcinoma; PRoNTo, Pattern Recognition for Neuroimaging Toolbox; RE, radiation encephalopathy; ReHo, regional homogeneity; ROC, receiver operating characteristic; rs-fMRI, resting-state functional MRI; SBM, surface-based morphometry; STG, superior temporal gyrus; SVM, support vector machine; VBM, voxel-based morphology.
