# Supplementary material for: Functional Connectivity Density for Radiation Encephalopathy Prediction in Nasopharyngeal Carcinoma
Source: Front Oncol. 2021 Jul 12;11:687127. doi: 10.3389/fonc.2021.687127 (PMC8311791; doi:10.3389/fonc.2021.687127)
Supplement: Supplementary file 1 [file DataSheet_1.pdf]

Supplementary Materials

1 Supplementary Tables

TABLE S1. Detailed chemotherapy data for patients with NPC

Chemotherapy regimens	Dose for each agent	Time for medication administration	Number of course	Duration (week)
**PF**				
5-FU (for neoadjuvant chemotherapy)	750-1000 mg/m^2,day1-5	day1-5, once every 3 weeks, three consecutive times	3	9
cisplatin (for neoadjuvant chemotherapy)	80-100mg/m^2	once every 3 weeks, three consecutive times	3	3
cisplatin (for concomitant chemoradiotherapy)				
- a single-pass regimen	80-100 mg/m^2	day 1-3/ 1st week, and day 1-3/ 5th week;	2	2
- weekly regimen	40 mg/m^2	once a week, seven consecutive times	7	7
**TP**				
docetaxel (for neoadjuvant chemotherapy)	75 mg/m^2	day1-5, once every 3 weeks, two consecutive times	2	6
cisplatin (for neoadjuvant chemotherapy)	80-100 mg/m^2	once every 3 weeks, two consecutive times	2	6
cisplatin (for concomitant chemoradiotherapy)				
- a single-pass regimen	80-100 mg/m^2	days 1-3/1st week, and days 1-3/5th week	2	2
- weekly regimen	40 mg/m^2	once a week, seven consecutive times	7	7
**TPF**				
5-FU (for neoadjuvant chemotherapy)	500-750 mg/m^2,day 1-5	day1-5,once every 3 weeks, two consecutive times	3	9
docetaxel (for neoadjuvant chemotherapy)	60 mg/m^2	once every 3 weeks, two consecutive times	3	9

cisplatin (for neoadjuvant chemotherapy)	60 mg/m^2	once every 3 weeks, three consecutive times	3	9
cisplatin (for concomitant chemoradiotherapy)				
- a single-pass regimen	80-100 mg/m^2	days 1-3/1st week, and days 1-3/5th week	2	2
- weekly regimen	40 mg/m^2	once a week, seven consecutive times	7	7
**GP**				
gemcitabine (for neoadjuvant chemotherapy)	1000 mg/m^2, day 1,8	day1-5,once every 3 weeks, two consecutive times	3	9
cisplatin (for neoadjuvant chemotherapy)	80-100 mg/m^2	once every 3 weeks, two consecutive times	3	9
cisplatin (for concomitant chemoradiotherapy)				
- a single-pass regimen	80-100 mg/m^2	days 1-3/1st week, and days 1-3/5th week	2	2
- weekly regimen	40 mg/m^2	once a week, seven consecutive times	7	7

Note: NPC, Nasopharyngeal carcinoma; RE, radiation encephalopathy; TPF, docetaxel, cisplatin and fluorouracil; TP, docetaxel and cisplatin; PF, cisplatin and fluorouracil; GP, gemcitabine and cisplatin

TABLE S2. The top 20 ranked brain regions represented 43.3% of the total weights of prediction.

Rank	%W	Brain region
1	2.80	Cingulum_Ant_L
2	2.72	Cuneus_L
3	2.54	Temporal_Pole_Sup_R
4	2.51	SupraMarginal_L

5	2.50	Angular_L
6	2.43	Temporal_Pole_Sup_L
7	2.33	Temporal_Sup_L
8	2.26	Cerebelum_4_5_R
9	2.19	Precuneus_R
10	2.18	Amygdala_L
11	2.17	Occipital_Mid_L
12	2.16	Cerebelum_Crus1_R
13	2.16	Frontal_Sup_Orb_R
14	2.10	Frontal_Inf_Oper_R
15	1.76	Occipital_Sup_R
16	1.76	Cuneus_R
17	1.71	Frontal_Inf_Tri_R
18	1.69	ParaHippocampal_R
19	1.67	Postcentral_R
20	1.67	Frontal_Sup_R

Abbreviation:L, left; Sup, superior; R, right; dor, dorsal; triangle, triangular; Inf, Inferior; Operc, Opercularis; ACG, anterior cingulum; TPO, temporal pole; AMYG, amygdala; ANG, angular; CUN, cuneus; MOG; middle occipital gyrus; SMG, supramarginal gyrus; STG, superior temporal gyrus; SOG, superior occipital gyrus; PCUU, precuneus; PoCG, postcentral gyrus; SFG, superior frontal gyrus; IFG, inferior frontal gyrus; ORB, orbital; PHG, parahippocampus; CRBL, cerebellum；%W，The percentage of total normalized weights that each brain region explains.

2 Supplementary scripts

2.1 Scripts of lFCD

```
clear;clc;
V_mask=spm_vol('fBrainMask_05_61x73x61.nii');
% [V_mask_val, ] = spm_read_vols(V_mask);
V_mask_val = spm_read_vols(V_mask);
nao_qu_yu_ti_shu =[];
GFDC = zeros(61,73,61);
mian_6 = [-1,0,0;
           1,0,0;
           0,-1,0;
           0, 1,0;
           0, 0,-1;
           0, 0,1];
% dian_2 = [-1,-1,-1;
%           -1,-1,0;
%           -1,-1,1;
%           -1,0,-1;
%           -1,0,0;
%           -1,0,1;
%           -1,1,-1;
%           -1,1,0;
%           -1,1,1;
%           0,-1,-1;
%           0,-1,0;
%           0,-1,1;
%           0,0,-1;
%           0,0,0;
%           0,0,1;
%           0,1,-1;
%           0,1,0;
%           0,1,1;
%           1,-1,-1;
%           1,-1,0;
%           1,-1,1;
%           1,0,-1;
%           1,0,0;
%           1,0,1;
%           1,1,-1;
%           1,1,0;
%           1,1,1;]

for sub=1:1
    %V=spm_vol(sprintf('c%dcosub%d.nii',tissue,sub));
    V=spm_vol('Filtered_4DVolume.nii');
    [V1, xyz] = spm_read_vols(V);
    i=0;
    for x=1:61
        x
        for y=1:73;

            for z=1:61

                tmpx=x;
                tmpy=y;
                tmpz=z;
```

```
        if(V_mask_val(x,y,z) > 0)

            flag = zeros(61,73,61);

            src=zeros(230,1);
            for t=1:230
                src(t,1) = V1(x,y,z,t);
            end
            index=0;
            stk=[];

            while 1
                for l = -1:1
                    for m= -1:1
                        for n =-1:1

                            if((tmpx+l~=x)&&(tmpy+m~=y)&&(tmpz+n~=z))
                                continue;
                            end
                            if(flag(tmpx+l,tmpy+m,tmpz+n) == 1)
                                continue;
                            end
                            if(V_mask_val(tmpx+l,tmpy+m,tmpz+n) > 0 )
                                dst = zeros(230,1);
                                for p=1:230
                                    dst(p,1) =
V1(tmpx+l,tmpy+m,tmpz+n,p);
                                end
                                R = corrcoef(src,dst);
                                if(R > 0.6)
                                    %GFDC(x,y,z) = GFDC(x,y,z)+1;
                                    %GFDC(l,m,n) = GFDC(l,m,n)+1;
                                    index = index+1;
                                    stk=[stk;[tmpx+l,tmpy+m,tmpz+n]];
                                    flag(tmpx+l,tmpy+m,tmpz+n) = 1;
                                end
                            end

                        end
                    end
                end
                if(index ==0)
                    break;
                else
                    tmpx=stk(1,1);
                    tmpy=stk(1,2);
                    tmpz=stk(1,3);
                    index=index-1;
                    stk=stk(2:end,:);
                    GFDC(x,y,z) = GFDC(x,y,z)+1;
                    GFDC(tmpx,tmpy,tmpz) = GFDC(tmpx,tmpy,tmpz)+1;
                end

            end
```

```
                end

            end
        end
    end

end
V(1).fname = 'GFDC.nii';
spm_write_vol(V(1),GFDC);
```

2.2 Scripts of gFCD

```
clear;clc;
tic
V_mask=spm_vol('fBrainMask_05_61x73x61.nii');
V_mask_val = spm_read_vols(V_mask);
nao_qu_yu_ti_shu =[];
GFDC = zeros(61,73,61);
num=0;
V=spm_vol('Filtered_4DVolume_li.nii');
[V1, xyz] = spm_read_vols(V);
i=0;
tic
for x=1:61
    x
    for y=1:73
        for z=1:61
            if(V_mask_val(x,y,z) > 0)
                src=zeros(180,1);
                for t=1:180
                    src(t,1) = V1(x,y,z,t);
                end
                linearInd = sub2ind(size(V_mask_val), x, y, z);
                num = num + 1;
                all_cell{num} = {linearInd,src};

            end
        end
    end
end

jieguo=[];
num
data = zeros(180,num);
for i=1:num
    i
    data(:,i)=all_cell{1,i}{1,2};
end

R = corrcoef(data);
toc
R(find(isnan(R)==1))=0;
R(R>0.6) = 1;
R(R<1) = 0;
sum_end = sum(R,2);
```

```
for i=1:num
    [x,y,z] = ind2sub(size(V_mask_val),all_cell{1,i}{1,1});
    GFDC(x,y,z) = sum_end(i,1);
end

V(1).fname = 'GFDC_501.nii';
spm_write_vol(V(1),GFDC);
pwl='finished'

clear;
toc
```
